# Chinese herbal fumigant and lotion for postoperative complication in surgical wound of anal fistula: A protocol for a systematic review and meta-analysis

**DOI:** 10.1097/MD.0000000000022095

**Published:** 2020-09-04

**Authors:** Liying Zhu, Sisi Ma, Chunhua Jia, Bei Zhang, Zimi Ma, EunHee Park

**Affiliations:** a School of Chinese Medicine; b School of Nursing, Beijing University of Chinese Medicine; c Beijing Guangji Traditional Chinese Medical Hospital, Beijing, China; d School of Physical Therapy, Taegu Science University, Taegu, South Korea.

**Keywords:** anal fistula, Chinese herbal fumigant and lotion, meta-analysis, postoperative complication, protocol, systematic review

## Abstract

**Background:**

Surgery is the most common and effective therapy for anal fistula, while the postoperative complication, such as pain, edema, pruritus, turgescence, and exudation in surgical wound, can have serious impact on wound healing and patients’ quality of life. Chinese herbal fumigant and lotion have been commonly used in postoperative treatment and achieved satisfied effect in China. However, clinical evidence-based literature of Chinese herbal fumigant and lotion for postoperative anal fistula is not sufficient. This protocol is described for a systematic review to investigate the beneficial effects.

**Methods:**

A systematic search will be conducted in database involving PubMed, the Cochrane library, Embase, Web of Science, Google Scholar, SinoMed, China National Knowledge Infrastructure(CNKI), VIP, Wanfang Database, CiNii(National Institute of Informatics), and KISS(Koreanstudies Information Service System) from inceptions to December 31, 2019. We will include randomized controlled trials (RCT) regarding Chinese herbal fumigant and lotion in the treatment of complication in surgical wound of anal fistula. Quality of included RCTs will be assessed according to the Cochrane Handbook 5.1.0. GRADE will be used to assess the quality of evidence. The summary results will be pooled using the random-effects model or fixed-effects model according to the heterogeneity of included studies.

**Results:**

After peer-review, the study will be disseminated in scientific journals and conferences.

**Conclusion:**

This systematic review will provide evidence for the efficacy of Chinese herbal fumigant and lotion for curing postoperative complication of anal fistula. In addition, it might provide suggestions for Chinese medicine clinical practice or guideline.

**PROSPERO registration:**

CRD42020164975.

## 1 Introduction

Anal fistula, also referred to as the fistula-in-ano, is an anorectum disease characterized as an abnormal communication between the anal glands and the perianal skin,^[1]^ which could cause chronic drainage from the anal area where usually a small opening near the anus with surrounding granulation tissue. The drainage can include stool, pus, or blood.^[2]^

The main etiology is perianal abscesses and suppurations. It mainly manifests as a variety of symptoms, such as discomfort, occasional pain, fecal incontinence, impaired quality of life, and work incapacity.^[3]^

Anal fistula is common in human beings and can occur at any age, but mainly in male adults, which can be ascribed to excessive secretion of sex hormone.^[4]^ There are significant differences in the number of patients with anal fistula here and abroad, accounting for 1.67% to 3.6% in China, and 8% to 25% in foreign countries. Domestically, the peak age of anal fistula is 20 to 40 years old and the ratio of male to female is 5-6:1.^[5]^ Recently, the incidence of anal fistula is increasing annually in China, which significantly affects patients’ quality of life.

Surgery such as simple fistulotomy remains one of the most effective therapies. The main objective of surgery for anal fistula is to eradicate the fistula tract while maintaining anal continence.^[6]^ Surgery remains one of the most effective therapies of a non-Crohn's anal fistula, the aim being to cure the fistula while at the same time preserving anal sphincter function.^[7]^ As the major cause of anal fistula is cryptoglandular infection, abscess formation is usual. Proper manipulations, such as curettage and drainage of blind sinuses, abscess cavities, and accessory tracts are the key for successful treatment.^[8]^

Anal fistula surgery can be performed under general, spinal, or local anesthesia as inpatient^[9]^ and the main reason for this is the concern about lack of postoperative pain control and associated problems.^[10]^ Compared to the surgical wound on other position, the healing process of the surgical wound of anal fistulotomy is much slower because of the presence of stool within the wound.^[11]^ Besides, the surgical wound infection with pain, pruritus, turgescence, and exudation is hard to healing and easy to occur. Therefore, it is of great necessity to shorten the wound healing time and repair contaminated wounds after surgery.^[12]^

Today, various medications have been studied for postoperative analgesia as suppositories, local anesthesia, or oral preparations. Yet the search for a suitable combination still continues.^[13]^ In addition to the routine postoperative treatment and western medicine, Chinese herbal fumigant and lotion have good pertinence to postoperative problems of anal fistula. Traditional Chinese medicine (TCM) plays an important role in maintaining health for Asian people. TCM has attracted the most attention for western countries in these years because of its reliable therapeutic efficacy and fewer side effects. Recently, fumigation-washing therapy on the surgical wound with Chinese herbal fumigant and lotion has been widely used for postoperative treatment and achieved satisfied effect.^[14]^ However, despite extensive clinical practice, clinical evidence-based literature of Chinese herbal fumigant and lotion for postoperative anal fistula is not sufficient. Therefore, this protocol is a preparation for gather recent research advances to provide evidence for clinical and public health specialist.

The review will address with reference to participants, interventions, comparators, and outcomes (PICO). All participants diagnosed with anal fistula and accepted the surgical treatment will be concerned. Interventions involving any Chinese herbal fumigant and lotion for the treatment of postoperative complications of anal fistula are eligible. Routine pharmacotherapy such as potassium permanganate, anti-inflammatory, and analgesic can be used together with Chinese herbs. Trials using Chinese herbs combining with microwave irradiation are also included. The control interventions include no treatment, placebo such as water or physiological saline, routine pharmacotherapy or other conventional treatments that are same as been used in the experimental group. The primary outcomes included clinical efficacy, score of pain measured by VAS, edema, and exudation. Secondary outcomes included healing time of surgical wound, function of anal sored by Wexner, rate of recurrence, adverse events, and quality of life.

## 2 Methods

### 2.1 Registration

This protocol has been registered with the PROSPERO registry of the University of York. The registration number is CRD42020164975.

This systematic review protocol will follow the guidelines of Preferred Reporting Items for Systematic Reviews and Meta-Analyses (PRISMA-P). Ethical approval was not necessary.

### 2.2 Eligibility criteria

#### 2.2.1 Types of studies

Randomized controlled trials (RCTs) will be included without restriction of publication type or language.

#### 2.2.2 Types of participants

All participants diagnosed with anal fistula and accepted the surgical treatment will be concerned. There will be no restrictions on gender, age, ethnicity, nationality, economic status, or education.

#### 2.2.3 Types of interventions

##### 2.2.3.1 Experimental interventions

Interventions involving any Chinese herbal fumigant and lotion for the treatment of postoperative complications of anal fistula are eligible. Routine pharmacotherapy such as potassium permanganate, anti-inflammatory, and analgesic can be used together with Chinese herbs. Trials using Chinese herbs combining with microwave irradiation are also included.

##### 2.2.3.2 Comparator interventions

The control interventions include no treatment, placebo such as water or physiological saline, routine pharmacotherapy, or other conventional treatments that are same as been used in the experimental group. If other kinds of Chinese herbal fumigant and lotion are used as comparator intervention, the study will be excluded.

#### 2.2.4 Types of outcome measures

##### 2.2.4.1 Primary outcomes

The primary outcomes included clinical efficacy, score of pain measured by VAS, edema, and exudation.

##### 2.2.4.2 Secondary outcomes

Secondary outcomes included healing time of surgical wound, function of anal sored by Wexner, rate of recurrence, adverse events, and quality of life.

### 2.3 Search strategy

#### 2.3.1 Electronic searches

A comprehensive search for eligible studies will be performed in database involving PubMed, the Cochrane library, Embase, Web of Science, Google Scholar, SinoMed, China National Knowledge Infrastructure(CNKI), VIP, Wanfang Database, CiNii(National Institute of Informatics), and KISS(Koreanstudies Information Service System) from inceptions to December 31, 2019. We will not apply any language restrictions. Search strategy of PubMed is presented in Table 1.

**Table 1:**
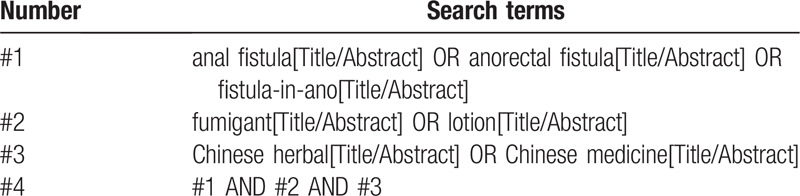
Search strategy for PubMed.

#### 2.3.2 Searching other resources

Relevant documents will also be retrieved, such as medical textbooks, clinical handbooks t, relevant conference proceedings, references list of eligible studies, and gray literatures. Meanwhile, experts in relevant fields will be contact to obtain important information that cannot be found from the retrieval.

### 2.4 Data collection and analysis

#### 2.4.1 Selection of studies

**Figure 1:**
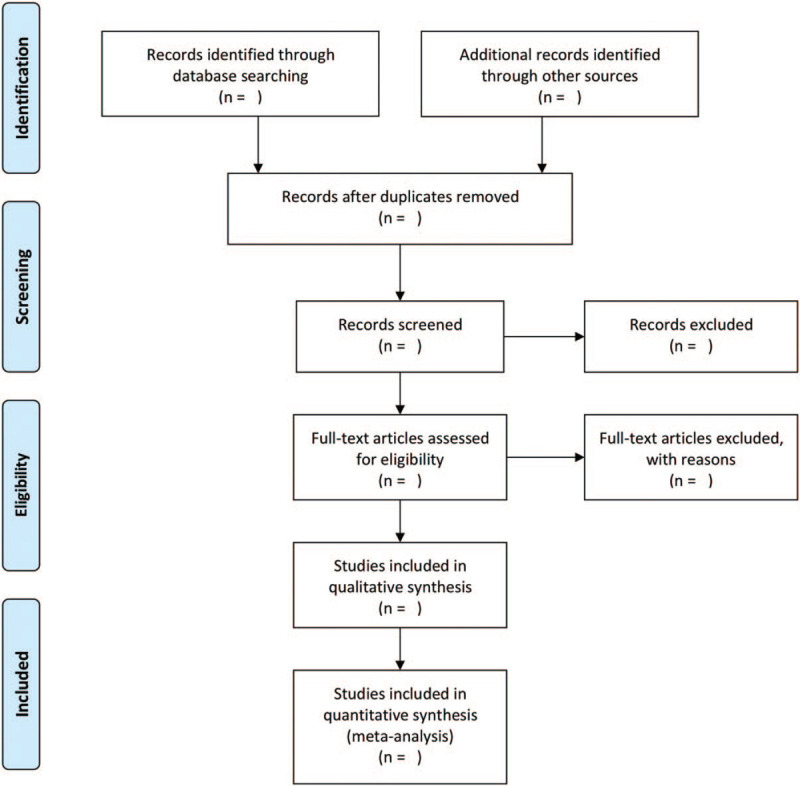
PRISMA flow diagram of the screening process.

The title and abstract of each record retrieved will be screened in EndNote X8 by two reviewers (LZ and BZ) independently to identify relevant studies according to eligibility criteria. Duplicate references will be removed at the same time. And then, full-text of all potentially relevant studies will be obtained and reviewed for further assessment. Disagreements will be resolved by discussion or consultation of a third reviewer (SM). The final literature will then be determined after reading all papers, discussion within group, and contacting the author to learn about the details of the study (Fig. 1). A final list will be developed with Microsoft Excel 2016 to collect relevant information.

#### 2.4.2 Data extraction and management

Two reviewers (ZM and EP) will independently extract information using a predetermined data form from the studies that met the inclusion criteria, including general information (title, authors, year of publication); details of study (aim, design, inclusion and exclusion criteria, method of randomization, and allocation); study participants (age, gender, sample size, classification of anal fistula, surgical method); intervention characteristics (type, duration, dose, follow-up time points, compliance); outcome (primary and secondary outcomes, time points, method of outcome assessments, blinding of outcome assessment, adverse effects). When information is missing, wrong or unclear, corresponding author of original study will be contacted. Any disagreement will be resolved by consensus or consultation with a third part.

#### 2.4.3 Risk of bias assessment

Two independent reviewers (ZL and ZB) will separately assess the risk of bias using the tool introduced in the Cochrane Handbook for systematic reviews of interventions (V.5.1.0).^[15]^ If consensus cannot be reached, the third reviewer (MS) will be consulted. Seven risks of bias domains will be assessed: sequence generation (selection bias); allocation concealment (selection bias); blinding of participants and personnel (performance bias); blinding of outcome assessment (detection bias); incomplete outcome data (attrition bias); selective outcome reporting (reporting bias); and other bias. The results of the assessment are low risk, unclear, and high risk. Inconsistencies can be resolved by discussing within the group or contacting the author to clear the details when necessary.

#### 2.4.4 Measures of treatment effect

The estimate of the effect for dichotomous outcome is represented by relative risk (RR) with 95% confidence interval (95% CI). Measurement data will be expressed as mean difference (MD) with 95% CI. When the same outcome is measured in a variety of ways, we will use the standardized mean difference (SMD) with 95% CI to express the size of the intervention effect.

#### 2.4.5 Dealing with missing data

With regard to missing data or clarification, we will attempt to obtain required information by contacting the corresponding author.

#### 2.4.6 Assessment of heterogeneity

The *χ*^2^ test is used to assess statistical heterogeneity between the results included in the study. In addition, the heterogeneity is quantitatively determined by using the *I*^2^ statistic value, which ranges from 0% to 100%. If *P* < 0.1of *χ*^2^ test or *I*^2^ > 50%, it is considered that there is statistically significant heterogeneity. Potential clinical heterogeneity will be assessed by pre specified subgroup analysis and sensitivity analysis.

#### 2.4.7 Assessment of publication bias

When 10 or more RCTs are included in meta-analysis, we will use a funnel plot to examine publication bias. In addition, we will test asymmetry using Egger test for continuous outcomes and Harbord modified test for dichotomous outcomes.

#### 2.4.8 Data synthesis

RevMan 5.3 software (The Cochrane Collaboration, Oxford, England) will be used to perform meta-analysis. More than one trial will be combined to estimate intervention effect when studies examine the same intervention and outcomes with comparable methods. The continuous data analyzed through the inverse variance method and dichotomous data analyzed through the Mantel-Haenszel method will be conducted. All data are expressed with 95% CI. The heterogeneity will be evaluated through *I*^2^ and *P* value. The fixed-effect model will be utilized to combine data when *I*^2^ ≤ 50% or *P* ≥ 0.1. However, when statistical heterogeneity exists, the random-effect model will be utilized to provide a more conservative estimate of effect.^[15]^

#### 2.4.9 Subgroup analysis

If the heterogeneity of the included studies is large, subgroup analyses will be performed on the basis of different interventions, controls, durations of treatment, and outcome measures. Adverse effects will be tabulated and assessed with descriptive techniques.

#### 2.4.10 Sensitivity analysis

Sensitivity analysis will be performed to evaluate the robustness of the results and to eliminate the impact of low-quality studies, provided there is significant heterogeneity after subgroup analysis and input data validation. After the low-quality study is removed, the meta-analysis will be performed again. The results of these two meta-analyses will be compared and then we will decide whether to exclude low-quality studies based on sample size, evidence strength, and impact on aggregated effective size. However, if all the included studies are at a high risk of bias, sensitivity analysis will not be conducted.

### 2.5 Ethics and dissemination

Ethical approval is not required because no primary and individual patient data are collected. In addition, findings from this systematic review will be disseminated through conference presentations and peer-reviewed scientific publications according to the PRISMA guidelines.

### 2.6 Quality of evidence

The quality of the research is evaluated by using the Grading of Recommendations Assessment, Development and Evaluation (GRADE) approach.^[15]^ According to the approach, the evidence is classified as high, moderate, low, very low quality based on the risk of bias, inconsistency, indirectness, imprecision, and publication bias. This step will be conducted through the online guideline development tool (GDT, http://gdt.guidelinedevelopment.org/). It is assumed that the quality of the evidence is the highest at first and gradually decreases according to the deficiencies of the study.

## 3 Discussion

Studies have shown that Chinese herbal fumigant and lotion can effectively alleviate the postoperative symptoms of anal fistula (mainly pain, edema and hard healing surgical wounds). Nevertheless, there is no English version of the systematic evaluation of that.

This is the first systematic review and meta-analysis to assess the efficacy of CHM for postoperative symptoms of anal fistula. We hope that it will provide more convincing evidence to help clinicians make decisions when dealing with anal fistula patients after surgery.

However, this systematic review has several limitations. The interventions of CHM vary from study to study. This may result in significant clinical heterogeneity.

## Acknowledgments

Thanks all the people for giving great advice on this article, especially Anorectal doctor Zhihao Deng and PhD candidate Ning Liang.

## Author contributions

SM, BZ and ZM contributed to the conception of the study. The manuscript protocol was drafted by LZ and revised by CJ and SM. The search strategy was developed by all the authors and will be performed by LZ, SM and BZ, who will also independently screen the potential studies, extract data from the included studies, assess the risk of bias, and complete the data synthesis. LZ will arbitrate in cases of disagreement and ensure the absence of errors. All authors approved the publication of the protocol.

**Conceptualization:** SM, BZ, ZM.

**Formal analysis:** LZ, SM, BZ.

**Software:** SM, EP.

**Supervision:** CJ.

**Writing – original draft:** LZ.

**Writing – review & editing:** CJ, SM.
